# Pulmonary Sequestration in the Guise of Pneumonia: Clues to a Hidden Entity

**DOI:** 10.7759/cureus.105628

**Published:** 2026-03-21

**Authors:** Siddharth Garg, Pramod K Sharma, Roshan Chanchlani, Aman Kumar, Shreya Rai, Suramya Jain, Ujjawal Khurana

**Affiliations:** 1 Pathology and Laboratory Medicine, All India Institute of Medical Sciences, Bhopal, Bhopal, IND; 2 Pediatric Surgery, All India Institute of Medical Sciences, Bhopal, Bhopal, IND; 3 Radiodiagnosis, All India Institute of Medical Sciences, Bhopal, Bhopal, IND

**Keywords:** adolescent medicine, anomalous systemic arterial supply, congenital lung malformations, lower lobe lung lesion, pulmonary sequestration

## Abstract

Pulmonary sequestration is an important yet often overlooked congenital lung malformation in adolescent medicine characterized by dysplastic, non-functioning pulmonary tissue that lacks communication with the tracheobronchial tree and derives its blood supply from an anomalous systemic artery. We report the case of a 12-year-old female patient referred to our institute with a six-month history of intermittent fever, cough, and chest pain, initially managed as a case of right-sided pneumonia elsewhere. Contrast-enhanced computed tomography of the thorax revealed a cavitary soft tissue lesion in the right lower lobe supplied by a systemic artery arising from the descending thoracic aorta with pulmonary venous drainage and no bronchial communication, establishing the diagnosis of intralobar pulmonary sequestration. Surgical resection was performed, and histopathological examination demonstrated cystically dilated bronchi and bronchioles with chronic inflammation, fibrosis, bronchiolization, and thick-walled systemic-type arteries, confirming the diagnosis. This case emphasizes the importance of considering pulmonary sequestration in patients with recurrent lower lobe infections and highlights the integrated role of imaging, histopathology, and pediatric surgical management in establishing the diagnosis, guiding definitive treatment, and preventing complications.

## Introduction

Pulmonary sequestration is a congenital lung malformation in which a segment or lobe of dysplastic, non-functioning lung tissue lacks communication with the tracheobronchial tree and receives an anomalous systemic arterial supply independent of the normal pulmonary circulation [1]. It accounts for a small proportion of congenital lung malformations and shows distinctive anatomical and vascular features [2].

It is classified into intralobar sequestration (ILS), which lies within the normal lung pleura and drains into the pulmonary veins, and extralobar sequestration (ELS), which is separate from the lung with its own pleural covering and drains into systemic veins.

Diagnosis relies on imaging modalities such as chest radiography and contrast-enhanced computed tomography (CECT) to identify the lesion and its anomalous systemic arterial supply, along with histopathological confirmation. Clinically, pulmonary sequestration may present with recurrent cough, fever, and hemoptysis, although some cases remain asymptomatic. Due to recurrent infection and impaired bronchial drainage, pulmonary sequestration may mimic pneumonia [3].

Reporting of this case is important due to its presentation and diagnostic challenges, highlighting the need for awareness among clinicians and pathologists. We present a case of pulmonary sequestration to emphasize its clinical, radiological, and pathological significance.

## Case presentation

**Figure 1:**
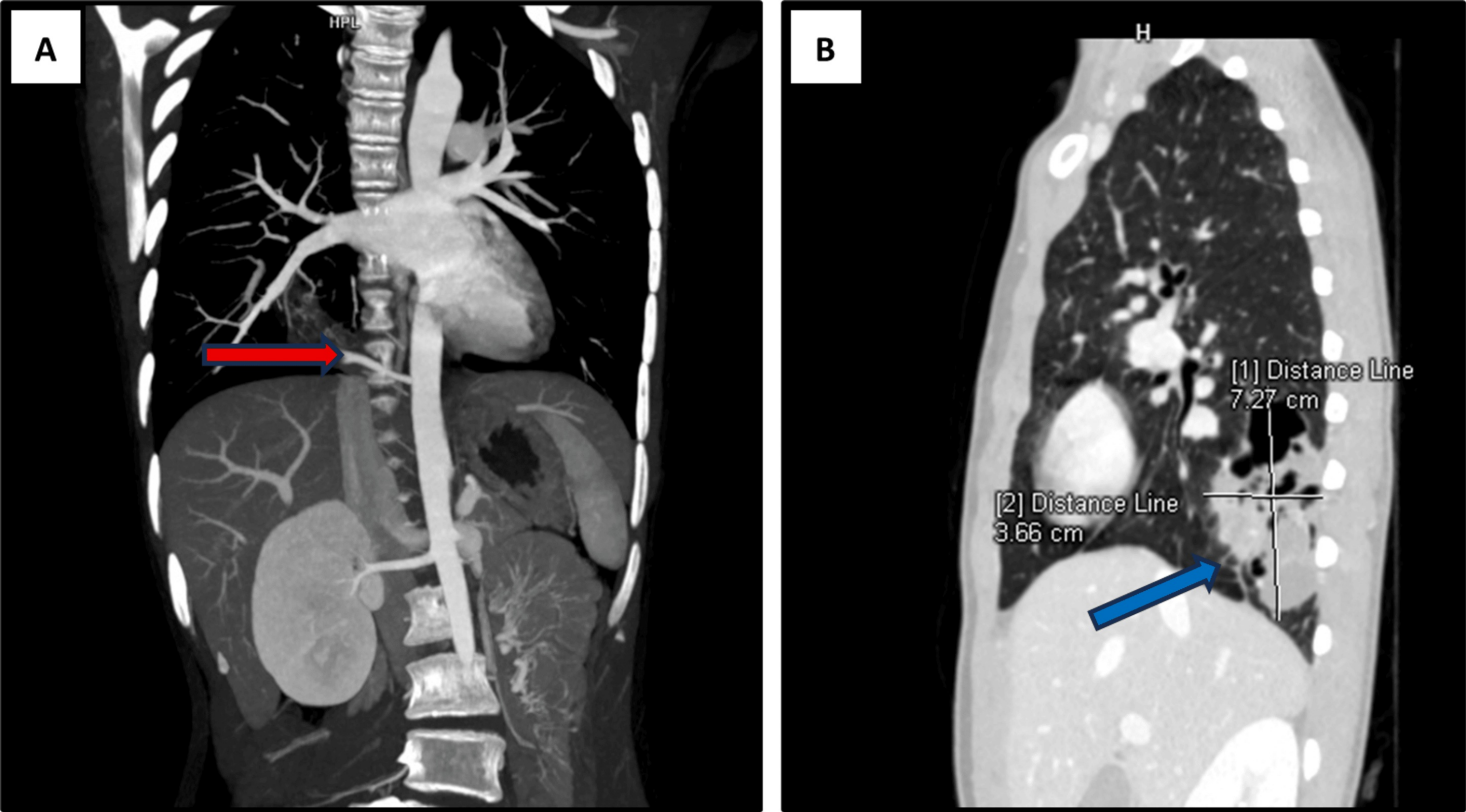
(A) CT angiographic image of the coronal section of the chest and abdomen demonstrating an anomalous systemic arterial supply (red arrow) arising from the descending thoracic aorta and coursing towards a well-defined lesion in the posterior basal segment of the right lower lobe. (B) Sagittal section of the lung window of CECT of the chest showing the sequestrated lung segment (blue arrow) measuring approximately 7.2×3.6 cm and showing no obvious communication with the tracheobronchial tree CT: computed tomography; CECT: contrast-enhanced computed tomography

**Figure 2:**
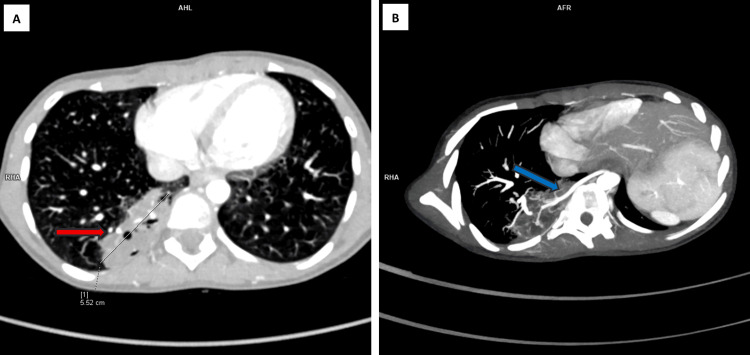
CECT angiographic images of the chest and upper abdomen. (A) CECT (axial section) demonstrating a cavitary lesion in the posterior basal segment of the right lower lobe (red arrow) measuring approximately 5.5 cm lacking any obvious bronchial communication. (B) CT angiographic reconstruction showing the anomalous systemic arterial feeder (blue arrow) arising from the descending thoracic aorta supplying the lesion CECT: contrast-enhanced computed tomography; CT: computed tomography; AHL: axial high-level view; RHA: right-handed acquisition; AFR: arterial flow reconstruction

A 12-year-old female patient, previously asymptomatic, was referred to our institute with a six-month history of intermittent fever and cough associated with chest pain, without dyspnea. She was admitted for further examination and evaluation. Respiratory examination revealed reduced air entry in the right lower lobe, while the remainder of the physical examination was unremarkable. She was initially managed as a case of right-sided pneumonia elsewhere. A CECT was eventually performed (Figure 1A-1B and Figure 2A-2B) which showed a multilobulated, cavitary soft tissue lesion in the posterior right lower hemithorax supplied by the descending thoracic aorta and draining into the inferior pulmonary vein, with no bronchial communication. The remaining lungs, mediastinum, pleura, heart, abdomen, and spine were unremarkable. The demonstration of an anomalous systemic arterial supply with pulmonary venous drainage of the sequestered pulmonary segment pointed towards the diagnosis of intralobar pulmonary sequestration.

Subsequently, the patient was planned for surgical correction by video-assisted thoracoscopic surgery (VATS) and underwent VATS-assisted right lower lobectomy. Intraoperatively, an ILS measuring approximately 6×5 cm was identified, supplied by a separate feeding artery (approximately measuring 5 mm) arising from the descending thoracic aorta, with venous drainage into the pulmonary vein. The specimen was sent for pathological examination. Postoperatively, the patient remained vitally stable, tolerated oral feeds well, and was discharged with an intercostal drain in situ, with instructions for regular follow-up.

**Figure 3:**
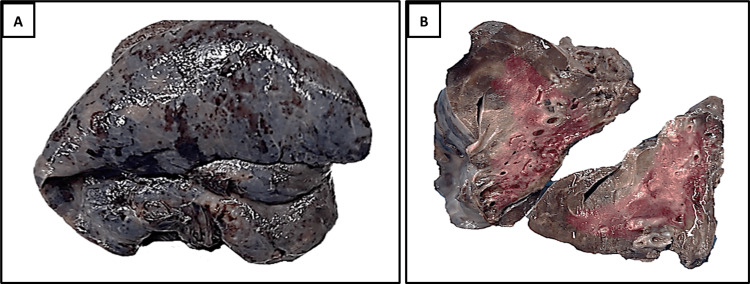
(A) External surface of the right lower lung lobe with congested pleural surface. (B) Cut surface of the right lower lung lobe showing cystically dilated bronchi with consolidated, congested, and hemorrhagic lung parenchyma

On gross examination (Figure 3A-3B), the resected right lower lobe specimen measured 8×5.5×4 cm and exhibited a grey-brown external surface. The cut surface showed multiple cystically dilated bronchi with features of bronchiectasis and surrounding consolidated, congested, and hemorrhagic lung parenchyma.

**Figure 4:**
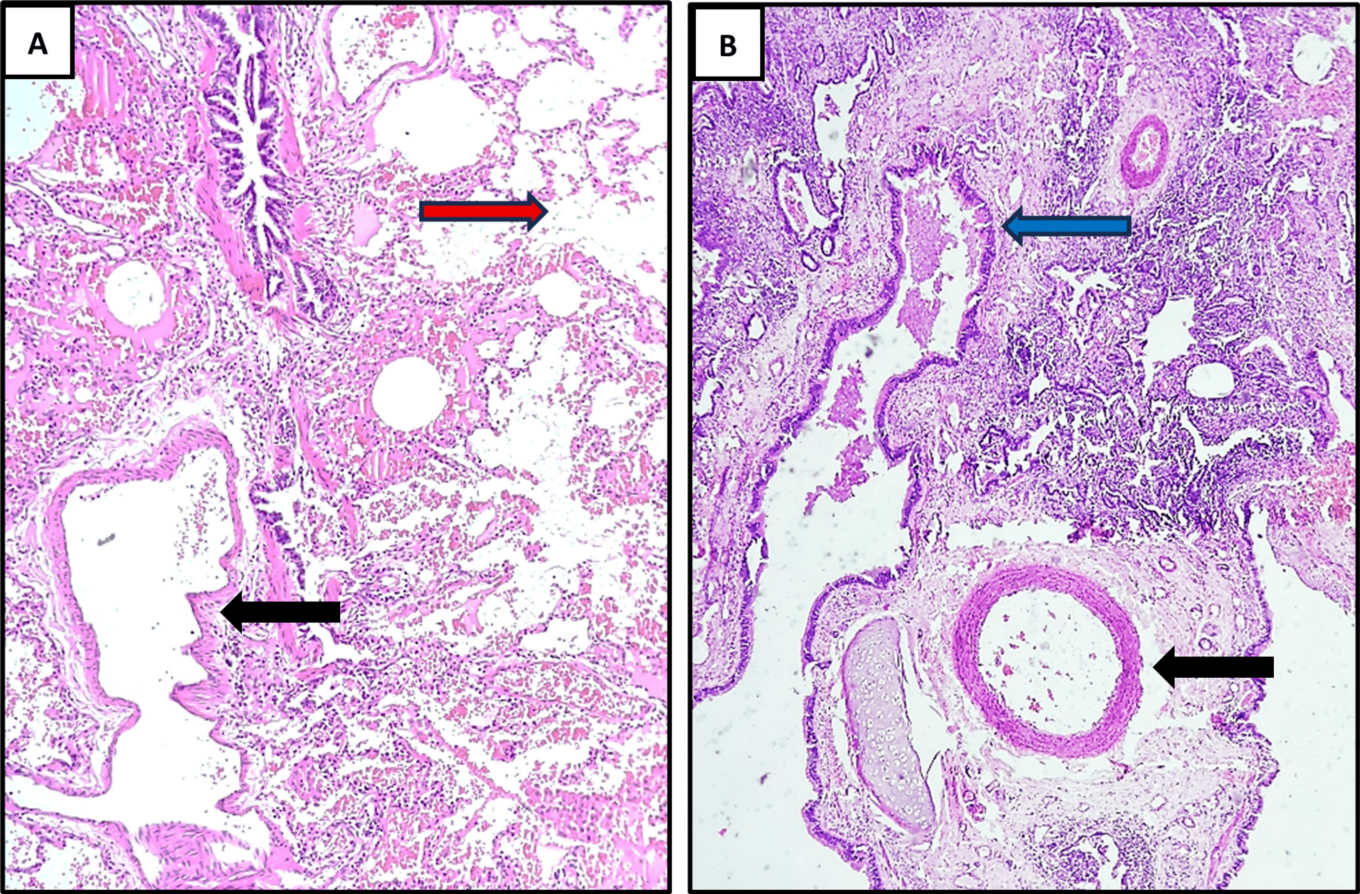
Histopathological features of intralobar pulmonary sequestration. (A) Photomicrograph (H&E stain, ×40) revealing a dilated blood vessel (black arrow) with smooth muscle wall and adjacent cystically dilated alveoli (red arrow) filled with mild inflammation and hemorrhage, set within the disorganized lung parenchyma. (B) Photomicrograph (H&E, ×40) showing a lung parenchyma with cystically dilated bronchus (lined by respiratory epithelium and presence of well-formed cartilage plate in its wall) filled with intraluminal mucin (blue arrow) and the presence of prominent thick-walled vascular channel (black arrow) H&E: hematoxylin and eosin

**Figure 5:**
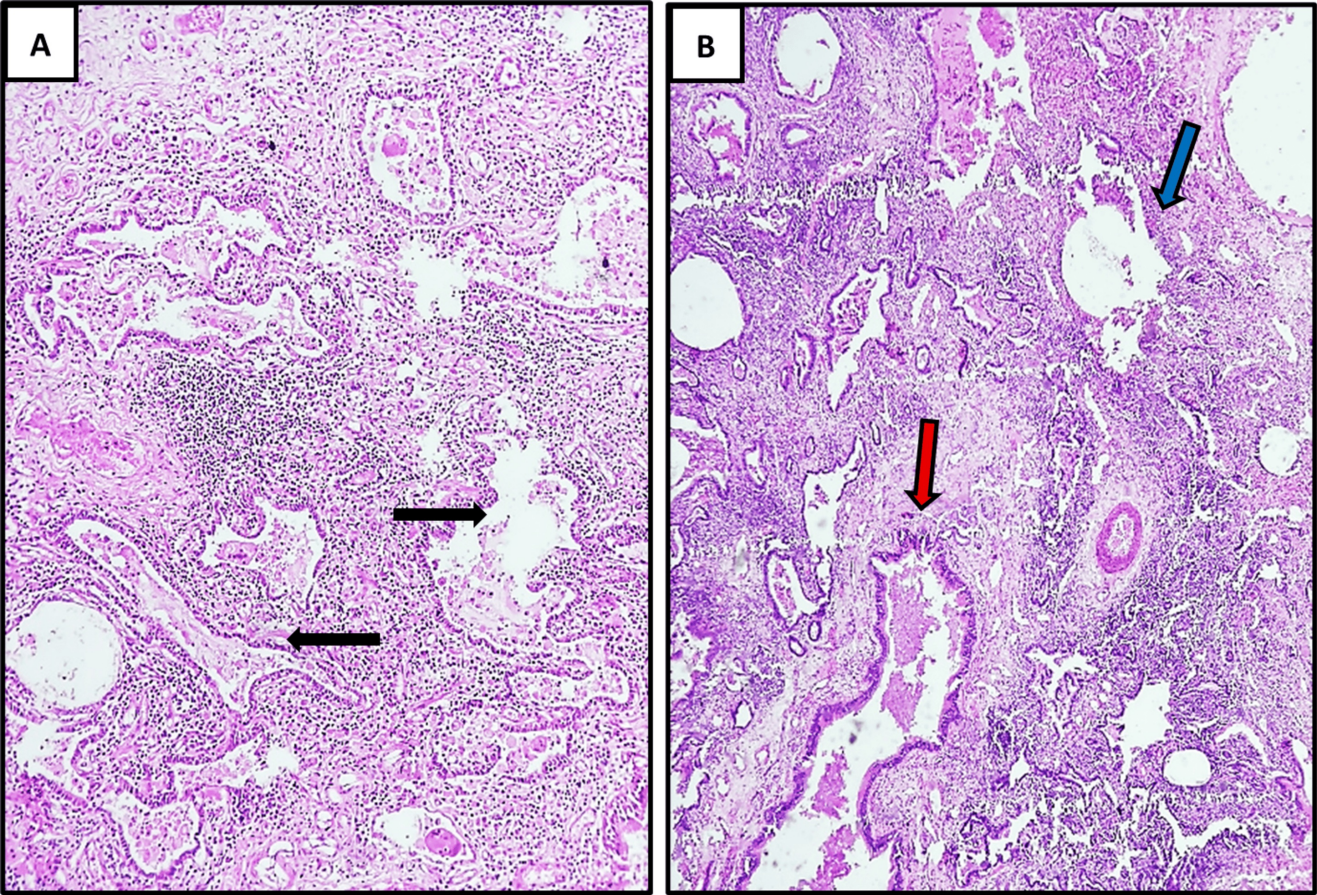
Histopathological features of intralobar pulmonary sequestration with evidence of pneumonia-like features. (A) Photomicrograph (H&E, ×100) demonstrating a lung parenchyma showing evidence of pneumonia-like features. There is evidence of bronchiolization of alveoli (black arrows) with intraluminal mucinous secretions and foamy macrophages embedded within the densely inflamed lung parenchyma. (5) Photomicrograph (H&E, ×40) showing cystically dilated bronchiole (red arrow) lined by respiratory epithelium and dilated alveolus (blue arrow) with luminal mucin stasis and adjacent lung parenchyma showing dense inflammation, hemorrhage, and thick-walled vascular channels along with peri-alveolar, peri-bronchiolar, and peri-vascular fibrosis H&E: hematoxylin and eosin

Subsequent histopathological examination (Figure 4A-4B and Figure 5A-5B) revealed cystically dilated bronchi and bronchioles lined by respiratory epithelium and dilated alveoli filled with mucin and foamy macrophages. Focal ulceration, fibroblastic proliferation, chronic inflammatory infiltrates, fibrosis, and evidence of bronchopneumonia were also noted. Alveoli showed collapse, hemorrhage, and interstitial fibrosis, with evidence of bronchiolization. Thick-walled arteries unaccompanied by bronchi were also identified. Overall, the gross and histopathological findings, correlated with clinical and radiological features, were consistent with intralobar pulmonary sequestration.

## Discussion

Pulmonary sequestration or bronchopulmonary sequestration is a rare congenital pulmonary malformation accounting for approximately 0.15-6.4% of all congenital pulmonary anomalies [4]. It is defined by the presence of dysplastic, non-functioning lung tissue that lacks communication with the tracheobronchial tree and derives its blood supply from an anomalous systemic artery [5]. Based on pleural investment, pulmonary sequestration is classified into ILS, which shares the visceral pleura of the adjacent lung, and ELS, which is enclosed within its own pleura. ILS is the more common subtype, comprising nearly 75% of cases, as is seen in our case [6].

Although several pathogenetic theories exist, the most widely accepted hypothesis proposes the formation of an accessory lung bud arising from the primitive foregut during embryogenesis, which acquires systemic arterial supply. The timing of this aberrant budding relative to pleural development determines the subtype, with early formation resulting in ILS and later development leading to ELS [7].

Pulmonary sequestration shows a marked predilection for the lower lobes, with nearly 97% of cases occurring in this location, and a two- to threefold higher incidence in the left lower lobe compared to the right; however, in contrast to this typical distribution, our case involved the right lower lobe [8]. Identification of an aberrant systemic arterial supply remains the cornerstone of diagnosis [9]. Most feeding vessels arise from the thoracic or abdominal aorta, although rarer sources have been described [10].

The clinical presentation of pulmonary sequestration varies according to subtype. ELS is frequently detected prenatally or in infancy and is often associated with other congenital anomalies, whereas ILS typically presents later in life, sometimes remaining asymptomatic until adulthood. When symptomatic, ILS most commonly manifests with recurrent pulmonary infections, cough, hemoptysis, fever, or chest pain [11].

Imaging plays a pivotal role in diagnosis. While ultrasound and magnetic resonance imaging (MRI) are valuable in prenatal and pediatric settings, CECT angiography remains the diagnostic modality of choice in adolescents and adults due to its ability to accurately delineate the aberrant arterial supply and venous drainage. The diverse radiologic appearance of ILS often leads to misdiagnosis as pneumonia, lung abscess, or neoplasm, underscoring the importance of vascular assessment [12].

The largest retrospective analytical study known to us by Wei and Li [13], comprising 2,625 cases of pulmonary sequestration in China (1998-2008), demonstrated a marked predominance of ILS (83.95%) over ELS (16.05%). The majority of lesions involved the lower lobes, particularly the left posterior basal segment (66.43%), followed by the right posterior basal segment (20.16%). Radiologically, the study described variable presentations, including mass-like, cystic, or cavitary lesions, while our case presented as a cavitary lesion in the right lower lobe, initially mimicking pneumonia. These findings highlight the diverse and often misleading clinical and imaging spectrum of pulmonary sequestration. Table 1 gives a summary of the demographic, clinical, and radiological data from different case series in comparison to the present case.

**Table 1:** Summary of the demographic, clinical, and radiological data from different case series in comparison to the present case ILS: intralobar sequestration; ELS: extralobar sequestration

Study	Sample size	Type (ILS/ELS)	Left lobe involvement	Main symptoms	Imaging findings	Source of anomalous supply
Wei and Li (2011) [13]	2625	84% cases/16% cases	71.5% cases	Cough, fever, hemoptysis	Mass lesion (46.33%), cystic lesion (28.57%), cavitary lesion (11.57%)	Thoracic aorta (76.6%), abdominal aorta (18.5%), intercostal artery (2%)
Sun and Xiao (2015) [8]	72	92.8% cases/7.2% cases	66.7% cases	Cough, hemoptysis	Soft tissue opacity (86.1%), cystic lesion (20.8%), cavitary lesion (13.9%)	Thoracic aorta (86.1%), abdominal aorta (6.9%)
Alsumrain and Ryu (2018) [6]	32	81% cases/19% cases	56% cases	Asymptomatic, cough	Mass/consolidation (61%), hyperlucency (42%), cystic changes (23%)	Thoracic aorta (54%), abdominal aorta (23%), celiac axis (11%)
Polaczek et al. (2017) [14]	25	88% cases/12% cases	52% cases	Infection, hemoptysis	Cystic changes/solid mass lesion	Thoracic aorta
Wang et al. (2023) [15]	97	90.7% cases/9.3% cases	77.3% cases	Cough, hemoptysis	Mass lesion (50.5%), cystic lesion (20.6%), cavitary lesion (10.3%)	Thoracic aorta (87.4%), abdominal aorta (10.5%)
Present case	1	ILS	Right lower lobe	Fever, cough	Cavitary lesion	Thoracic aorta

Surgical resection remains the definitive treatment for pulmonary sequestration, particularly in symptomatic patients and in asymptomatic ILS due to the risk of life-threatening hemoptysis [16]. VATS has increasingly replaced open thoracotomy owing to reduced morbidity and favorable outcomes [17]. Endovascular embolization has emerged as an alternative therapy; however, concerns regarding incomplete occlusion and recurrence limit its widespread use [18]. Management of ELS remains individualized, with observation recommended for asymptomatic cases and surgery reserved for those developing complications [19].

Pulmonary sequestration commonly enters the differential diagnosis of pneumonia because patients often present with recurrent fever, cough, chest pain, and radiologic consolidation involving the lower lobes. Owing to its variable appearance as a persistent consolidation, cystic or cavitary lesion, or mass-like opacity, it may also be confused with a lung abscess or focal bronchiectasis. Other important differentials include congenital pulmonary airway (cystic adenomatoid) malformation, bronchogenic cyst, and congenital lobar emphysema, all of which may present as congenital lung lesions with overlapping imaging features. In cases of ELS with abdominal extension, retroperitoneal tumors may also be considered in the differential diagnosis [20].

This case underscores the importance of maintaining a high index of suspicion for pulmonary sequestration in patients presenting with recurrent lower lobe infections or hemoptysis. Demonstration of anomalous systemic arterial supply on imaging, complemented by pathologic confirmation and coordinated pediatric surgical management, is essential for accurate diagnosis and timely treatment, thereby preventing delayed intervention and potential complications.

## Conclusions

Pulmonary sequestration is a rare congenital lung malformation that may present with recurrent infections, fever, cough, or hemoptysis and should be considered in the differential diagnosis of such presentations. This case highlights intralobar pulmonary sequestration presenting as a right lower lobe cavitary lesion that clinically and radiologically mimicked pneumonia, resulting in an initial misdiagnosis. Accurate preoperative identification of the anomalous systemic arterial supply on imaging is essential for definitive diagnosis and surgical planning. Surgical resection remains the treatment of choice to prevent complications by enabling safe isolation and division of aberrant feeding vessels with histopathology confirming the diagnosis. Recognizing this entity is essential, as early diagnosis can avoid delays in appropriate surgical intervention and reduce the risk of recurrent infections and other potential complications. Further studies are warranted to elucidate the etiopathogenesis of pulmonary sequestration and to better define the role of embryologic, infectious, genetic, and environmental factors in its development.
